# Extracellular vesicles from dental pulp mesenchymal stem cells modulate macrophage phenotype during acute and chronic cardiac inflammation in athymic nude rats with myocardial infarction

**DOI:** 10.1186/s41232-024-00340-7

**Published:** 2024-05-28

**Authors:** Elena Amaro-Prellezo, Marta Gómez-Ferrer, Lusine Hakobyan, Imelda Ontoria-Oviedo, Esteban Peiró-Molina, Sonia Tarazona, Pedro Salguero, Amparo Ruiz-Saurí, Marta Selva-Roldán, Rosa Vives-Sanchez, Pilar Sepúlveda

**Affiliations:** 1 Regenerative Medicine and Heart Transplantation Unit, Health Research Institute Hospital La Fe, Avda. Fernando Abril Martorell 106, Valencia, 46026 Spain; 2 Department of Analytical Chemistry, Faculty of Chemistry, University of Valencia, Valencia, 46100 Spain; 3 Hospital Universitari I Politècnic La Fe, Valencia, 46026 Spain; 4 Department of Applied Statistics and Operations Research and Quality, Universitat Politècnica de València, Valencia, 46022 Spain; 5 Department of Pathology, University of Valencia, Valencia, 46010 Spain; 6 Centro de Investigación Biomédica en Red Enfermedades Cardiovasculares (CIBERCV), III Institute of Health, Madrid, Carlos Spain

**Keywords:** Extracellular vesicles, Mesenchymal stromal cells, Acute myocardial infarction, Inflammation, Macrophage

## Abstract

**Background/aims:**

Extracellular vesicles (EVs) derived from dental pulp mesenchymal stem cells (DP-MSCs) are a promising therapeutic option for the treatment of myocardial ischemia. The aim of this study is to determine whether MSC-EVs could promote a pro-resolving environment in the heart by modulating macrophage populations.

**Methods:**

EVs derived from three independent biopsies of DP-MSCs (MSC-EVs) were isolated by tangential flow-filtration and size exclusion chromatography and were characterized by omics analyses. Biological processes associated with these molecules were analyzed using String and GeneCodis platforms. The immunomodulatory capacity of MSC-EVs to polarize macrophages towards a pro-resolving or M2-like phenotype was assessed by evaluating surface markers, cytokine production, and efferocytosis. The therapeutic potential of MSC-EVs was evaluated in an acute myocardial infarction (AMI) model in nude rats. Infarct size and the distribution of macrophage populations in the infarct area were evaluated 7 and 21 days after intramyocardial injection of MSC-EVs.

**Results:**

Lipidomic, proteomic, and miRNA-seq analysis of MSC-EVs revealed their association with biological processes involved in tissue regeneration and regulation of the immune system, among others. MSC-EVs promoted the differentiation of pro-inflammatory macrophages towards a pro-resolving phenotype, as evidenced by increased expression of M2 markers and decreased secretion of pro-inflammatory cytokines. Administration of MSC-EVs in rats with AMI limited the extent of the infarcted area at 7 and 21 days post-infarction. MSC-EV treatment also reduced the number of pro-inflammatory macrophages within the infarct area, promoting the resolution of inflammation.

**Conclusion:**

EVs derived from DP-MSCs exhibited similar characteristics at the omics level irrespective of the biopsy from which they were derived. All MSC-EVs exerted effective pro-resolving responses in a rat model of AMI, indicating their potential as therapeutic agents for the treatment of inflammation associated with AMI.

**Supplementary Information:**

The online version contains supplementary material available at 10.1186/s41232-024-00340-7.

## Background

Myocardial infarction (MI) is a leading cause of mortality and morbidity [1, 2]. The implementation of reperfusion approaches has decreased the mortality rate; however, a large majority of patients who survive an acute MI (AMI) are at high risk of subsequent adverse left ventricular remodeling and heart failure [3, 4]. This risk is closely associated with a failure to repair and remodel the injured heart [5]. Cardiac repair following AMI involves a series of complex and orchestrated events [5]. One of the first events to occur is the inflammatory response [6, 7]. The ischemic event induces damage in cardiomyocytes and other cell types present in the heart, which release damage-associated molecular patterns (DAMPs) [8, 9]. Immune cells, mainly macrophages and neutrophils, recognize DAMPs and promote immune cell infiltration in the myocardium to clear damaged cells [7]. The clearance of apoptotic cells and damaged extracellular matrix leads to a reparative phase characterized by the resolution of inflammation, scar formation, and neovascularization [10]. Nevertheless, when the inflammatory process is exaggerated or prolonged, it can lead to improper healing, which promotes adverse remodeling and heart failure [11].

Macrophages play a pivotal role in the inflammatory process that occurs after AMI. They are responsible for clearing matrix debris and dead cells, and they are the primary effector cells in inducing the resolution of inflammation [12]. The populations of macrophages in the infarcted heart after AMI are highly heterogeneous [13], and their phenotype has recently been deciphered in the mouse model in the context of AMI [14]. Grossly, they can be classified into pro-inflammatory macrophages (M1-like) that act at the beginning of the inflammatory process, and pro-resolving macrophages (M2-like) that are involved in the final phase of inflammation. M2-like macrophages facilitate wound healing by inducing the proliferation of myofibroblasts and the generation of mature scar tissue [15].

Recent advances suggest that targeting the inflammatory process may lead to better outcomes in AMI, minimizing the risk of heart failure and mortality [16, 17]. Although some attempts have been made to treat inflammation after AMI [18–22], none has been successful, in part because of the complexity of the inflammatory process. Therefore, new therapeutic strategies are needed. In this context, therapies based on extracellular vesicles (EVs) derived from mesenchymal stem cells (MSCs) may become a plausible option to mitigate the adverse effects of MI.

The contribution of MSCs to cardiac repair has been assessed in several studies [18–20]. However, their beneficial effect has been related to their paracrine effect rather than a direct effect based on engraftment and differentiation into other cell types [21]. Among the paracrine signals of MSCs, one of the most notable are extracellular vesicles (EVs), which are membrane-bound particles released by cells that play an important role in intercellular communication in health and disease [22, 23]. We have previously demonstrated the ability of EVs derived from dental pulp MSCs (DP-MSC-EVs) to interact with various cardiac cells [24, 25]. MSC-derived EVs are loaded with bioactive molecules, including proteins, lipids, mRNA, and microRNAs, which can induce various responses in the receptor cells [26]. Several studies have shown that MSC-EVs recapitulate the immunomodulatory capacity of their parental cells, promoting the suppression of inflammatory processes and inducing tissue repair [27, 28].

In the context of AMI, EVs may stimulate beneficial effects related to cell survival and immunomodulation that favor tissue repair and regeneration. These effects translate into improved cardiac function [29]. However, to date, the production of MSC-EVs has several limitations and challenges that hinder their clinical use. These limitations include the heterogeneity of MSC biopsies, standardization and scalability of EV isolation processes, and extraction yields [30]. In this work, we aimed to evaluate whether EVs isolated from three independent biopsies of DP-MSCs have similar phenotypes and behave equally in terms of immunomodulation in the context of AMI. To this end, we evaluated the in vitro capacity of MSC-EVs to modulate the macrophage populations and their in vivo effect in a nude rat model of experimental myocardial infarction, focusing on changes in cardiac immune populations.

## Methods

### Cell culture

Human DP MSCs isolated from three different young healthy donors (10–25 years old) were immortalized by transduction with the lentiviral vector pLV-hTERT-IRES-hygro encoding human telomerase (Addgene, #85,140; Watertown, MA, USA). MSCs were cultured in Dulbecco’s modified Eagle’s medium (DMEM)-low glucose (Gibco, Thermo Fisher Scientific, Waltham, MA, USA) supplemented with 10% fetal bovine serum (FBS) and 10 µg mL-1 ciprofloxacin (Sigma-Aldrich, Saint Louis, MO, USA). To obtain MSC-EVs, MSCs were cultured for 48 h in an extraction medium consisting of DMEM with 20% EV-depleted FBS and antibiotics. EV-depleted FBS was prepared by ultracentrifugation of a mixture of the same volume of FBS and DMEM at 100,000 g for 16 h.

Primary cultures of monocytes were isolated from buffy coats of healthy donors after informed consent. Blood was diluted in Hank’s buffered salt solution (HBSS; Gibco, Thermo Fisher Scientific) and centrifuged at 3950 rpm for 9 min. The middle layer was collected, and peripheral blood mononuclear cells (PBMCs) were isolated by density gradient centrifugation in Histopaque®-1077 (Sigma-Aldrich). Monocytes were isolated from PBMCs by positive magnetic separation using Flow-Comp™ CD14 Dynabeads® (Invitrogen, Waltham, MA, USA). Isolated monocytes were cultured in Rosewell Park Memorial Institute (RPMI) medium (Gibco, Thermo Fisher Scientific) supplemented with 10% FBS and 10 µg/mL ciprofloxacin. Monocytes were differentiated into M1 or M2 macrophages by adding to the complete medium 5 ng/mL of recombinant human granulocyte–macrophage colony-stimulating factor (rhGM-CSF, Invitrogen) or 20 ng/mL of recombinant human macrophage colony-stimulating factor (rhM-CSF, Invitrogen), respectively, on days 0 and 3. On day 5, 10 ng/mL of lipopolysaccharide (LPS, Invitrogen) and 20 ng/mL of interferon γ (IFN-γ, R&D Systems, Minneapolis, MN, USA) were added to M1 macrophages, whereas 40 ng/mL of interleukin-4 (IL-4) and 20 ng/mL of IL-13 (both from PeproTech, London, UK) were added to M2 macrophages. During M1 differentiation, 2 × 10^9^ or 4 × 10^9^ particles/mL of MSC-EVs were added at day 0.

Human polymorphonuclear neutrophils (PMNs) were also isolated from buffy coats of healthy donors after informed consent. Blood was diluted in HBSS and centrifuged on a Lympholyte®-poly (Cedarlane®, Burlington, Canada) density gradient for 50 min at 450 g. The bottom monolayer was collected, and cells were washed by centrifugation in PBS at 400 g for 10 min. The pellet was resuspended in PBS and cells were cultured.

### Extracellular vesicle isolation

MSC-EVs were isolated from 200 mL of MSC extraction medium after 48 h of culture. To extract MSC-EVs, a combined method based on tangential flow filtration (TFF) followed by size exclusion chromatography (SEC) was used (TFF-SEC) [31]. Briefly, molecules > 100 kDa were concentrated using a tangential flow filter (HansaBioMed, Tallinn, Estonia) to a volume of 2 mL. Subsequently, the MSC-EVs contained in the medium were isolated on EV SEC columns (STEMCELL Technologies, Vancouver, Canada). MSC-EVs were resuspended in PBS for functional analysis and characterization by nanoparticle tracking analysis (NTA), dynamic light scattering (DLS), and asymmetrical flow field flow fraction (AF4).

We have submitted all relevant data of our experiments to the EV-TRACK knowledgebase (EV-TRACK ID: EV240019) [32].

### Western blotting

Samples were resuspended in RIPA buffer (1% NP40, 0.5% deoxycholate, 0.1% sodium dodecyl sulfate in tris-buffered saline [TBS]) (Sigma-Aldrich) supplemented with protease (Complete, Sigma-Aldrich) and phosphatase (PhosSTOP, Sigma-Aldrich) inhibitors. Equal amounts of samples were mixed with Laemmli sample buffer (BioRad, Hercules, CA, USA) and denatured at 95ºC for 5 min. Proteins were separated on SDS–polyacrylamide gels and transferred to polyvinylidene difluoride membranes (ImmobilonP; Millipore, Bedford, MA, USA), which were blocked with 5% non-fat dry milk in TBS. The primary antibodies used for MSC-EV characterization were as follows: anti-CD9 (dilution 1:500, [C-4] Sc-13118), anti-TSG101 (dilution 1:200, [C-2] Sc-7964), anti-CD81 (dilution 1:200, sc-7637), anti-ALIX (dilution 1:200, [3A9] sc-53538) (all from Santa Cruz Biotechnology, Santa Cruz, CA, USA), anti-Calnexin (dilution 1/500, Santa Cruz, H-70), and anti-HSP70 (dilution 1:500, 4876, Cell Signaling Technology, Danvers, MA, USA). For in vivo studies, the primary antibodies used were anti-GAPDH (dilution 1:1000, 5174, Cell Signaling Technology), anti-CX3CR1 (dilution 1:500, [1H14L7] 702,321, Thermo Fisher Scientific), anti-CCR2 (dilution 1:500, PA5-23,037, Thermo Fisher Scientific), anti-MSR1 (dilution 1:500, PA5-102,519, Thermo Fisher Scientific), and anti-*iNOS* (dilution 1:500, PA1-036, Thermo Fisher Scientific). Detection was performed using peroxidase-conjugated antibodies and SuperSignal™ West Femto substrate (Thermo Fisher Scientific). Reactions were visualized using GE Healthcare, Chicago, IL, USA, and quantified with ImageJ software (NIH, Bethesda, MD, USA).

### Nanoparticle tracking analysis

MSC-EV samples were resuspended in sterile PBS (Thermo Fisher Scientific). Samples were diluted to a concentration suitable for further analysis. EV size distribution and quantification were analyzed on the Nanosight NS3000 System (Malvern Panalytical, Malvern, UK).

### Transmission *electron* microscopy

Electron microscopy was performed as described [33]. Briefly, MSC-EVs were diluted in PBS, loaded onto Formwar carbon-coated grids, contrasted with 2% uranyl acetate, and finally examined on an FEI Tecnai G2 Spirit transmission electron microscope (FEI, Eindhoven, The Netherlands). Images were acquired using a Morada CCD Camera (Olympus Soft Image Solutions GmbH, Münster, Germany).

### Dynamic light scattering and asymmetric flow field-flow fractionation analysis

The size distribution of MSC-EVs, PDI value, and Z-potencial were measured by DLS using a Zetasizer Nano ZS (Malvern Instruments, Malvern, UK) equipped with a 633 He–Ne laser (operating at an angle of 173). All measurements were performed in triplicate in PBS at 25 °C.

### Single-particle fluorescence and interferometry imaging with ExoView®

ExoView® technology is based on the interferometric imaging of a single EV particle, enabling analysis of its size and protein profile. EVs are imaged on a silicon substrate chip, consisting of a matrix of printed dots with different antibodies. This can be combined with immunostaining using fluorophore-conjugated antibodies so that different markers can be characterized simultaneously in the same vesicle. The commercial ExoView® Human Tetraspanin Kit (NanoView Biosciences, Brighton, MA, USA), with antibodies attached to CD9, CD81, and CD63, was used to characterize the MSC-EVs. Briefly, isolated MSC-EVs were diluted 1:10 in the 1X Incubation Solution, and 50 µL of the diluted sample was added to a silicon chip deposited in a p-24 well. The samples were incubated with the chips overnight and washed 3 times in agitation using Solution A. The chips were then incubated on a shaking platform for 1 h with a fluorescent antibody mixture consisting of anti-CD81 (CF® 555), anti-CD9 (CF® 488A), and anti-CD63 (CF® 647) in a blocking solution and washed as indicated. Data and image acquisition was performed on the ExoView® R100 platform (NanoView Biosciences), and data analysis was performed with ExoView Analyzer 3.1.4 software.

### Proteomic analysis

Proteomic analysis of the MSC-EV biopsies was performed as described [34]. Briefly, 20 µg of a protein sample of each biopsy was treated with DL-dithiothreitol, iodoacetamide, and trypsin. Next, 4 μL of each sample was loaded for liquid chromatography and tandem mass spectrometry (LC–MS/MS). Peptides were analyzed on a nanoESI qQTOF (6600plus TripleTOF, ABSCIEX) mass spectrometer. ProteinPilot default parameters were used to generate peak lists directly from 6600 plus TripleTOF wiff files. The Paragon algorithm [35] of ProteinPilot v5.0 was used to search SwissProt (version 200,601) with the following parameters: trypsin specificity, cys-alkylation, taxonomy non-restricted and human taxonomy, and the search effort set to through with FDR analysis. Proteins showing unused scores > 1.3 were identified with confidence ≥ 95%.

Data were analyzed with String software. Proteins with an unused value > 1.3 were used for gene ontology (GO) analysis to detect enriched biological processes in the experimental groups. The results were represented graphically using the ggplot2 package [36] from R [37]. Venny diagrams were obtained using Venny 2.1.0 [37].

### Lipidomic analysis

Lipidomic analysis of the MSC-EV biopsies was performed using liquid chromatography (UPLC) coupled to a high-resolution mass spectrometer (MS) with an Orbitrap UPLC-QExactive Plus detector (UPLC-TOF/MS-Orbitrap QExactive Plus MS), available at the Analytical Unit of the Instituto de Investigación Sanitaria La Fe (IISLaFe, Valencia, Spain).

The chromatographic separation was performed using an Acquity UPLC CSH C18 (100 × 2.1 mm, 1.7 μm) column from Waters Corp. (Milford, MA, USA). The mobile phase in the positive ionization mode was acetonitrile/water (60:40, v/v) with ammonium formate (10 mM) (A) and isopropyl alcohol/acetonitrile (90:10, v/v) with ammonium formate (10 mM) (B), while the mobile phase in the negative ionization mode was acetonitrile/water (60:40, v/v) with ammonium acetate (10 mM) (A) and isopropyl alcohol/acetonitrile (90:10, v/v) with ammonium acetate (10 mM) (B). Data processing, peak picking, retention time alignment, and peak integration were performed using the LipidMSv3 R package [38] to obtain annotated lipids in the samples.

### MicroRNA sequencing and quantification

MicroRNA sequencing was performed with MSC-EVs from the three biopsies before and after the immortalization of DP-MSCs with hTERT (MSC-EVs derived from non-immortalized and immortalized MSCs). For this purpose, miRNAs from MSC-EVs were isolated using miRNeasy Mini Kit (Qiagen, Westburg BV, Leusden, The Netherlands). Libraries were prepared using the NEBNext® Small RNA Library Prep Set (Illumina®, E7330, San Diego, CA, USA). Briefly, 1–5 ng of RNA isolated from MSC-EVs was subjected to adaptor 3’ and 5’ ligation and first-strand cDNA synthesis. Library amplification was performed by PCR using indexed primers supplied in the kit. Libraries were analyzed using the Agilent Bioanalyzer to estimate the quantity and check size distribution and were sequenced on Illumina’s NextSeq2000 platform.

The quality of sequencing data, consisting of fasta files, was evaluated using FastQC [39]. To ensure the precision of subsequent analyses, we employed Atria software [40] for automatic adapter detection within the raw data. The detected adapters were then meticulously trimmed using the Cutadapt tool [41], configured for a quality threshold of 30, a minimum read length of 15, and a singular adapter removal per read. The reads were aligned against the human mature miRNA database (GECh38) sourced from miRNABase.org [42], utilizing bowtie2 [43]. After alignment, data processing included the exclusion of reads with a MAPQ score below 1, achieved through Samtools [44]. We then processed the alignment files to count the occurrence of each miRNA by its identifiers.

We selected those miRNAs that were present in at least two of the three samples. A secondary criterion retained miRNAs with cumulative counts surpassing 100 in at least 2 biopsies. Functional enrichment analysis was performed on the selected miRNAs using the GeneCodis platform [45]. Significant associations were demarcated based on adjusted *p* values with an established threshold of 0.05.

### Flow cytometry

Cells were washed and incubated with a blocking solution of PBS containing 1% of normal mouse serum for 10 min. Thereafter, cells were incubated with saturating amounts of fluorochrome-conjugated antibodies for 1 h at 4ºC and washed. Human antibodies used were as follows: anti-CD14-RPE (TUK4, Dako, Santa Clara, CA, USA), anti-CD163 PerCP-Cy (GHI/61, BD Biosciences, Franklin Lakes, NJ, USA), anti-CD80 APC (FUN-1, BD Biosciences), anti-CD86 V450 (L307.4, BD Biosciences), and anti-HLA-DR FITC (AC122, Miltenyi Biotec, Bergisch Gladbach, Germany) at concentrations recommended by the manufacturers. Samples were processed using a BD FACSCANTO II flow cytometer and results were analyzed using FlowJo® software (FlowJo LLC, BD, Franklin Lakes, NJ, USA).

### Real-time quantitative PCR

RNA was isolated from macrophages or heart tissue using the RNeasy Plus Mini Kit (Qiagen, Westburg BV, Leusden, The Netherlands). RNA was quantified spectrophotometrically using a NanoDrop ND-1000 (NanoDrop Technologies, Wilmington, DE, USA). cDNA was obtained by reverse transcription using the PrimeScript RT Reagent Kit (Takara, Kusatsu, Japan). RT-qPCR was performed in 384 well plates with the respective sense and anti-sense primers and TB Green® Premix Ex TaqTM (Tli RNase H Plus, Takara, Kusatsu, Japan). RT-qPCR was run on a Viia 7 PCR System (Applied Biosystems, Waltham, MA, USA). The primers used were as follows:

- *hGAPDH*:CCCCTCTGCTGATGCCCCCA (F) and TGACCTTGGCCAGGGGTGCT (R)
- hCD206:ACCTGCGACAGTAAACGAGG (F) and TGTCTCCGCTTCATGCCATT (R)
- *hCD163*:GCTCATCCCGTCAGTCATCC (F) and TCCCGGTATTGAATTTGGTGGA (R)
- *hALOX15*:TTCTATGCCCAAGATGCGCT (F) and TGCAGCCCGATTTCAGTGAT (R)
- *hCXCL10*:TGCAAGCCAATTTTGTCCACGTGT (F) and GCAGCCTCTGTGTGGTCCATCC (R)
- *hCXCL11*:TGTCTTTGCATAGGCCCTGGGGT (F) and AGCCTTGCTTGCTTCGATTTGGGA (R)
- *hCCL5*:TACATTGCCCGCCCACTGCC (F) and GGGTTGGCACACACTTGGCG (R)
- *rTnfα*:GATCGGTCCCAACAAGGAGG (F) and GCTTGGTGGTTTGCTACGAC (R)
- *riNos*:AGTCAACTACAAGCCCCACG (F) and GCAGCTTGTCCAGGGATTCT (R)
- *rGapdh*:GCATCTTCTTGTGCAGTGCC (F) and GATGGTGATGGGTTTCCCGT (R)
- *rCd274*:CTCCTCGCCTACAGGTAAGTC (F) and ACACCACTAAGGCAAGCAGG (R)
- *rMsr1*:CTGTGGGCCAGATGCTGAAA (F) and CCTGAACGGAGGAGCCATTT (R)
- *rAlox12*:ACTTGACTTGGATCGCCTCC (F) and AGTCCTCGGCAACATCGAAA (R)
- *rCx3cr1*:TCGTCCTGCCCTTGCTTATC (F) and GTCTCCGTCACACTAAGGGC (R)

### Measurement of cytokines by enzyme-linked immunosorbent assay

Cell culture supernatants were used to measure IL-10, IL-1β, and TNFα levels. The quantification of these cytokines was conducted using commercial ELISA kits (Invitrogen, Waltham, MA, USA).

### Efferocytosis

Isolated neutrophils were cultured in PBS for 24 h to induce apoptosis and then stained with Texas Red (APC, Thermo Fisher Scientific). Stained apoptotic neutrophils were co-cultured with differentiated macrophages (1:1 ratio) for 2 h. Monocytes differentiated to M1 were treated or not with MSC-EVs on day 0 of differentiation. Macrophages were washed after co-culture and stained with anti-human CD14 RPE TUK4, Dako). Flow cytometry was used to analyze the percentage of CD14 and APC double-positive cells, representing macrophages that had efferocytosed apoptotic neutrophils.

### Experimental in vivo model of acute myocardial infarction

#### Animals

Nude male rats weighing 200–250 g were purchased from Envigo (Indianapolis, Indiana, USA) and housed at the IISLa Fe animal housing facility under standard conditions. The number of animals included in the study was 30. All animal procedures were approved by institutional ethical and animal care committees.

#### Acute myocardial infarction procedure

AMI was performed by permanent ligation of the left anterior descending (LAD) coronary artery. Rats were anesthetized with a mixture of O_2_ and sevoflurane (Sevorane™, AbbVie Limited, Dublin, Ireland) and intubated. The infarcted area was visualized after ligation by the development of a pale color in the distal myocardium. Immediately after LAD ligation, 2.5 × 10^9^ MSC-EVs or saline were injected intramyocardially with two injections of 10 µL at two discrete locations of the infarct border zone with a Hamilton syringe. Rats were euthanized 7- and 21-days post-AMI.

#### Measurement of infarct size

Rats were perfused with 2% paraformaldehyde and hearts were extracted, dehydrated, embedded in paraffin, sectioned at 5-µm thickness, and mounted on glass slides. Infarct size was assessed using Masson’s trichrome staining. For this, sections were incubated in Bouin’s solution for 15 min at 56 °C, washed with running water, incubated with ferric hematoxylin for 5 min, washed again, and incubated with Biebrich scarlet-acid fuchsin. Shortly after, fuchsin was removed by gentle washing, and the slides were incubated with phosphomolybdic acid for 15 min, followed by anillin blue for 1 min. Finally, slides were incubated with 1% glacial acetic acid; washed with 70, 96, and 100% ethanol; incubated in xylol; and mounted. All staining chemicals and related reagents were from Sigma-Aldrich. Sections were observed using a Leica DM6000 inverted optical microscope (Leica Microsystems GmbH, Wetzlar, Germany), and the images were analyzed with ImageJ. Infarct size was measured in 8–12 transverse sections of 5 µm (1 slice each 200 µm of tissue) from apex to base and expressed as the mean of the percentage of the left ventricular fibrotic area of all slices from each heart.

#### Cytokine profiling

The cytokine profile of rat heart samples was assessed using the commercial Proteome Profiler Rat Cytokine Array kit (R&D Systems, Minneapolis, MN, USA). Cryopreserved heart samples were homogenized in a solution of PBS with cOomplete™ protease inhibitor (Sigma-Aldrich) and 1% Triton® X-100 and kept cold. Nitrocellulose membranes with the captured antibodies of interest were incubated in an Assay Solution for 1 h under agitation at room temperature. Simultaneously, the homogenized samples were incubated with 15 µL of the detection antibody cocktail at room temperature for 1 h. Subsequently, the sample/antibody mixtures were added to the membranes and allowed to incubate overnight at 4 °C shaking. The next day, the membranes were washed 3 times, for 10 min each, and incubated with 2 mL of a solution of Streptavidin-HRP in Assay Solution for 30 min at room temperature with shaking. The membranes were rewashed 3 times and developed with the reagent mix provided by the kit. Images were acquired on an Amershan Imager 600 (GE Healthcare, Piscataway, NJ, USA). Protein expression was determined by densitometry using ImageJ.

#### Immunofluorescence

For immunofluorescence staining, 5-µm-thick heart slices were blocked and permeabilized with a solution of 5% FBS and 0.1% Triton-X100 (Sigma-Aldrich) in PBS for 1 h at room temperature. Slices were incubated overnight in a humidified chamber at 4ºC with the following antibodies: rat anti-F4/80 (dilution 1:200, Abcam, ab6640, Cambridge, UK), rabbit anti-CD206 (dilution 1:200; Abcam, ab64693), rabbit anti-PDL1 (dilution 1:200, ABClonal A11273, Wuhan, China), rabbit anti-CX3CR1 (dilution 1:200, Thermo Fisher Scientific, 702,321), rabbit anti-CCR2 (dilution 1:50, Thermo Fisher Scientific, PA5-23,037), rabbit anti-MSR1 (dilution 1:200, Thermo Fisher Scientific, PA5-102,519), rabbit anti-ARG1 (dilution 1:200, Thermo Fisher Scientific, PA5-29,645), and rabbit anti-iNOS (dilution 1:50, Thermo Fisher Scientific, PA1-036). Thereafter, slices were washed with PBS 3 times (5 min each) and incubated with anti-rat IgG Alexa 555 (Invitrogen), and anti-rabbit IgG Alexa 488 (Invitrogen) secondary antibodies for 1 h at room temperature in a humidified dark chamber. Heart sections were washed and counterstained with 200 ng/mL DAPI (Thermo Fisher Scientific) for 10 min in the dark. Heart slices were finally washed and mounted with FluorSave™ reagent (CalbioChem, San Diego, CA, USA). The sections were visualized with a fluorescent Leica DM2500 microscope (Leica Microsystems). Final image processing and quantification were performed using ImageJ.

### Statistical analysis

Data are expressed as mean ± SD. The normality of the distribution was analyzed with the Kolmogorov–Smirnov test. Student’s *t* test was used for normal unpaired samples in between-group comparisons. One-way analysis of variance (ANOVA) with Tukey’s multiple comparisons test was used to compare the means of more than 2 normal groups. Kruskal Wallis with Dunn’s multiple comparisons test was used to compare the means of more than 2 non-normal groups. Analyses were conducted with GraphPad Prism 8 software (San Diego, CA, USA). Differences were considered statistically significant at *p* < 0.05 with a 95% confidence interval.

## Results

### Characterization of MSC-EVs and lipidomic analysis

**Fig. 1:**
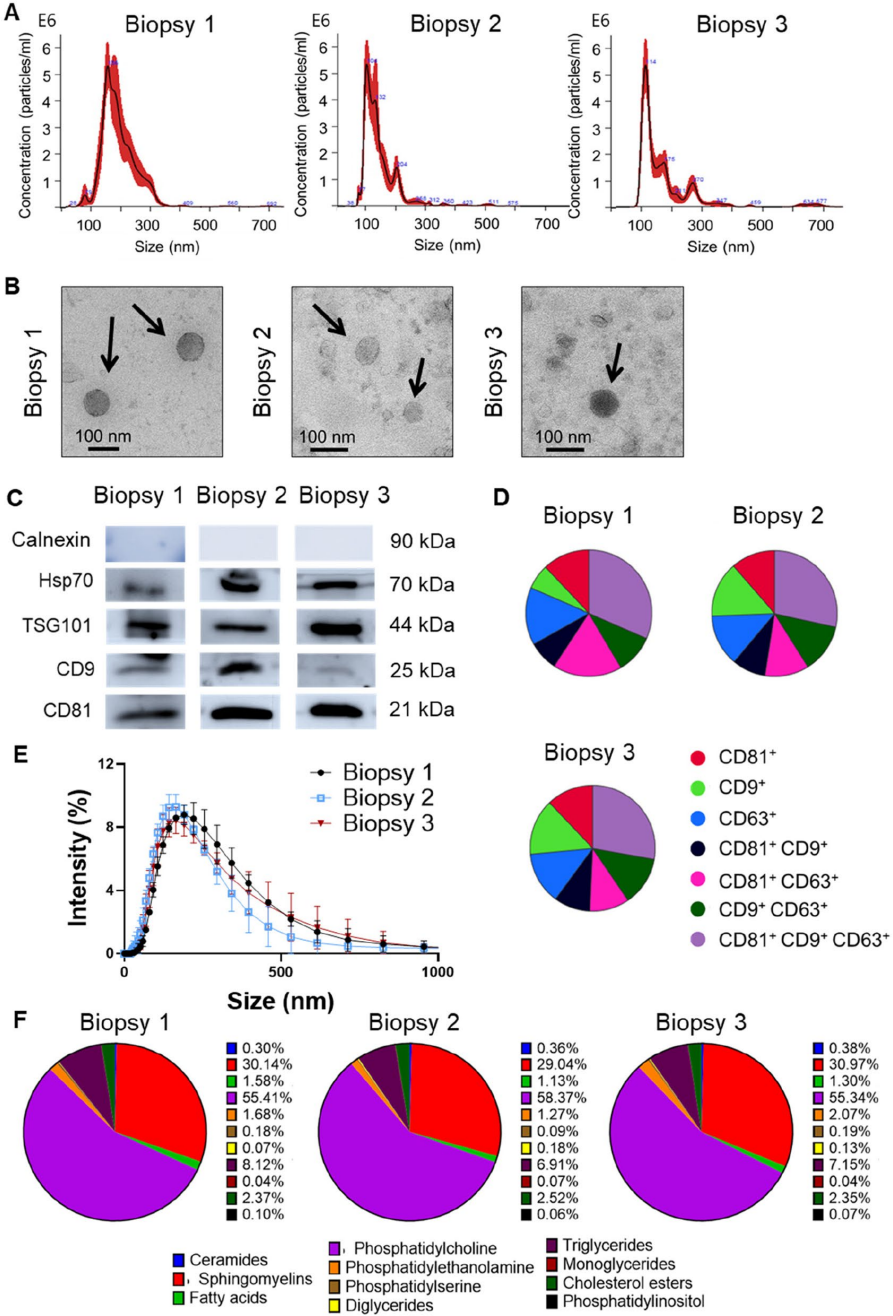
Characterization of extracellular vesicles (EVs) isolated from three different dental pulp mesenchymal stem cell (DP-MSC) biopsies. **A** Representative MSC-EV sizes and concentrations analyzed by NTA. **B** Representative electron microscopy images of isolated MSC-EVs. The black arrows indicate MSC-EVs. Scale bar = 100 nm. **C** Representative western blotting of Hsp70, Tsg101, CD9, CD81, and Calnexin protein expression. Calnexin protein expression was used as a negative control for MSC-EVs. **D** Representative nanoview graphs of CD63, CD81, and CD9 analysis. **E** DLS analysis of MSC-EVs representing the size distribution (*n* = 3). **F** Lipidomic analysis of MSC-EVs obtained from three DP-MSC biopsies. The different lipid groups present in the MSC-EVs are shown together with their proportion with respect to total lipids

MSC-EVs were isolated from the conditioned medium of human DP-MSCs. To overcome the difficulty of producing homogeneous batches of MSC-EVs in large quantities [46], primary MSCs were first immortalized with hTERT, as described [47] and MSC-EVs were isolated using TFF-SEC. To assess whether the therapeutic capacity of MSC-EVs is independent of biopsy, we isolated and compared MSC-EVs from three different DP biopsies. NTA revealed that the MSC-EV populations were 100–250 nm in size (Fig. 1A). Transmission electron microscopy analysis was consistent with the observed diameters measured by NTA and revealed a circular membranous structure (Fig. 1B). Western blotting showed that MSC-EVs expressed the typical exosome markers HSP70, TSG101, CD9, and CD81 (Fig. 1C). The presence of the tetraspanins CD63, CD81, and CD9 was demonstrated by Nanoview analysis, with a similar profile in all three biopsies (Fig. 1D). DLS validated the size of MSC-EVs (Fig. 1E) and revealed that their polydispersity index, indicating the degree of homogeneity of the EV population, was similarly independent of the biopsy (0.398 ± 0.004 in biopsy 1, 0.389 ± 0.07 in biopsy 2, and 0.415 ± 0.06 in biopsy 3). We also measured the *Z*-potential value of each sample of harvested MSC-EVs. This value describes the net electrical charge of the molecules present on the surface of the MSC-EVs and is a measure of colloidal stability and aggregation [48]. All biopsies showed similar *Z*-potential values, indicating stable formulations (− 11.23 ± 3.55 in biopsy 1, − 12.33 ± 2.9 in biopsy 2, and − 12.65 ± 1.72 in biopsy 3).

Lipidomic analysis was performed to identify the lipid groups in the three biopsies of MSC-EVs (Supplementary Table 1). The different lipid group proportions were almost identical in the MSC-EVs of the independent samples (Fig. 1F). Phosphatidylcholine was the major lipid group, representing 55–60% of the lipids, followed by sphingomyelins (~ 30%) and triglycerides (~ 7%). Thus, MSC-EVs derived from independent biopsies had the same lipid composition, characterized by the abundance of phosphatidylcholine.

### Proteomic and miRNA analysis indicate that MSC-EVs modulate inflammation-related biological processes

**Fig. 2:**
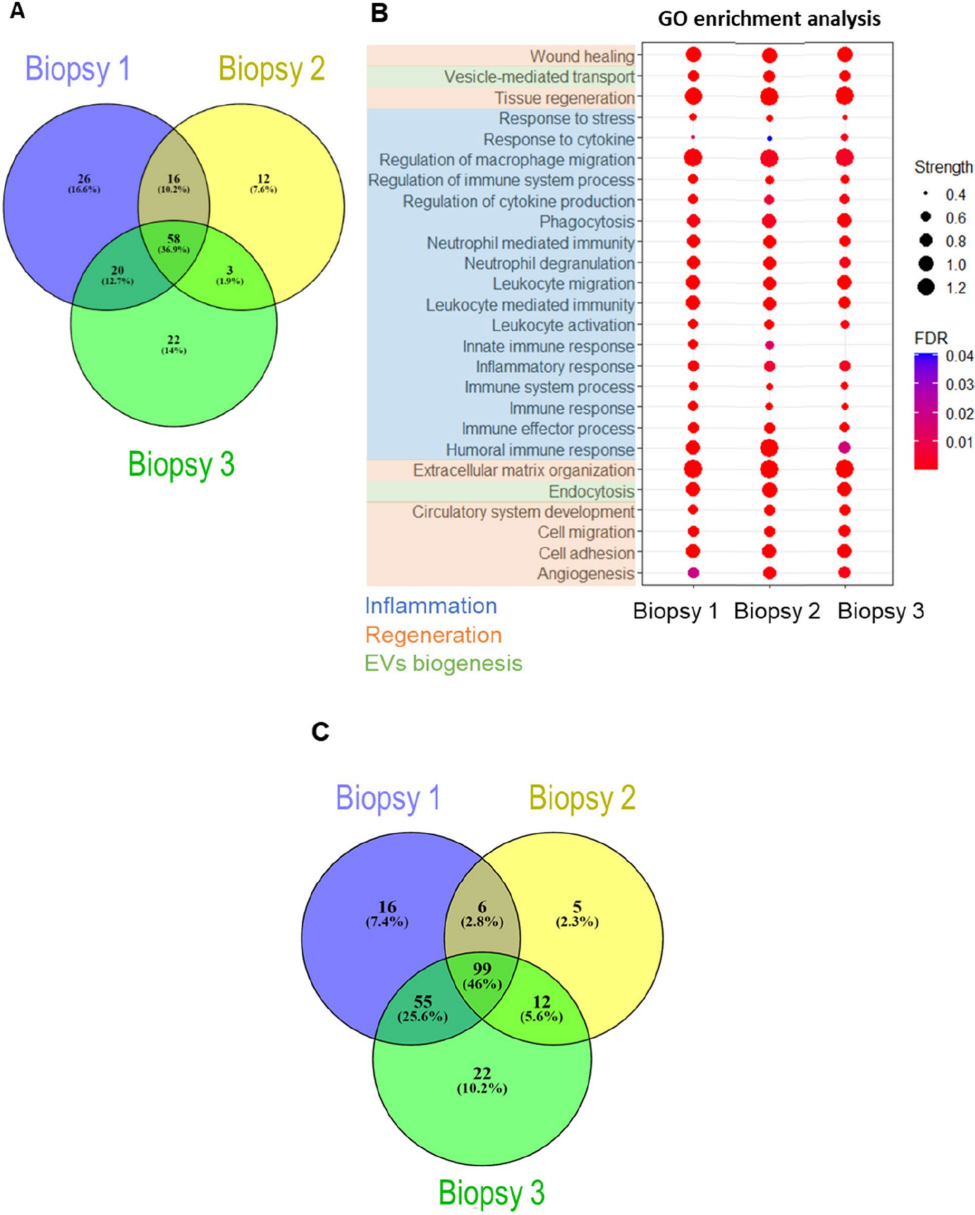
Graphical representation of proteomic and miRNAseq analysis of MSC-EVs. **A** Venn diagram of data from proteomic analysis of MSC-EVs isolated from three different biopsies. **B** Graphical representation of statistically significant biological processes in the three biopsies. FDR, false discovery rate. **C** Venn diagram of miRNAs detected in MSC-EVs from three biopsies

We next performed a comparative proteomic analysis of the three MSC-EV samples to explore the biological processes that can potentially be modulated by these MSC-EVs. Detected proteins with an unused value > 1.3 were selected from biopsies and compared using the Venny 2.1.0 (https://bioinfogp.cnb.csic.es/tools/venny/index.html) online web tool (Supplementary Table 2). Overall, 36.9% of the proteins were common to the MSC-EVs of the three biopsies. However, when considering the proteins that were common to two or three biopsies, the percentage increased to 61.7% (Fig. 2A). The protein content of MSC-EVs was used to analyze the potential involvement of these proteins in different GO biological processes using the String database. Most of the enriched biological processes were related to inflammation (Fig. 2B, blue), followed by processes related to tissue regeneration (green) and vesicle production and transport (orange). These data suggest that EV protein cargo potentially modulates processes related to the immune system.

We next performed microRNA sequencing on EV extracts from the three DP-MSC biopsies before and after immortalization with hTERT and checked that no major changes in miRNA expression had occurred due to telomerase transduction. The raw data was uploaded to the GEO database. We assessed the inter-biopsy variability by examining the similarity in miRNA content (Fig. 2C). In total, 176 miRNAs were identified in the EVs derived from MSC biopsy 1, 122 from biopsy 2, and 188 from biopsy 3. In total, 99 miRNAs were common to all three (Supplementary Table 3), accounting for 46% of the total miRNAs found, with 172 miRNAs (80% of the total) present in MSC-EVs from at least two biopsies. The results were filtered to obtain the most representative miRNAs in MSC-EVs. A total of 11 miRNAs showed more than 100 counts (hsa-miR-15b-5p, hsa-miR-16-5p, hsa-miR-21-5p, hsa-miR-29a-3p, hsa-miR-29c-3p, hsa-miR-30b-5p, hsa-miR-126-5p, hsa-miR-199a-3p, hsa-miR-199b-3p, hsa-miR-221-3p, hsa-miR-451a). The biological processes in which these miRNAs in the MSC-EVs were involved were studied, which revealed that a remarkable number of significant biological processes involving the most abundant miRNAs were related to immune response and inflammation (Table 1), consistent with the proteomic analysis [27].

**Table 1:** Significant biological processes in which the selected miRNAs are involved

GO reference	Biological processes	*P* value
GO:0030198	Extracellular matrix organization	2.01E − 05
GO:0071407	Cellular response to organic cyclic compound	2.67E − 05
GO:0030225	**Macrophage differentiation**	2.99E − 05
GO:0001776	**Leukocyte homeostasis**	4.01E − 05
GO:0042542	Response to hydrogen peroxide	5.12E − 05
GO:0001782	**B cell homeostasis**	6.86E − 05
GO:0043029	**T cell homeostasis**	7.26E − 05
GO:0006897	Endocytosis	9.12E − 05
GO:0030224	**Monocyte differentiation**	1.06E − 04
GO:0034097	**Response to cytokine**	1.11E − 04
GO:0030217	**T cell differentiation**	1.35E − 04
GO:0045766	**Positive regulation of angiogenesis**	3.77E − 04
GO:0002376	**Immune system process**	5.11E − 04
GO:0006955	**Immune response**	5.28E − 04
GO:0019221	**Cytokine-mediated signaling pathway**	5.93E − 04
GO:0006959	**Humoral immune response**	7.70E − 04
GO:0042060	**Wound healing**	7.70E − 04
GO:0007507	heart development	1.13E − 03
GO:0042981	**Regulation of apoptotic process**	1.61E − 03
GO:0001819	**Positive regulation of cytokine production**	3.52E − 03
GO:0006954	**Inflammatory response**	5.59E − 03
GO:0050729	**Positive regulation of inflammatory response**	6.20E − 03
GO:0002548	**Monocyte chemotaxis**	8.46E − 03
GO:0034599	Cellular response to oxidative stress	1.18E − 02

The table shows the Gene Ontology biological process reference (left), the name of the biological process, and the adjusted *p* value of each biological process. Some of the biological processes related to the immune system and inflammation are highlighted in bold

## MSC-EVs promote the polarization of M1 macrophages towards an M2-like phenotype in vitro

**Fig. 3:**
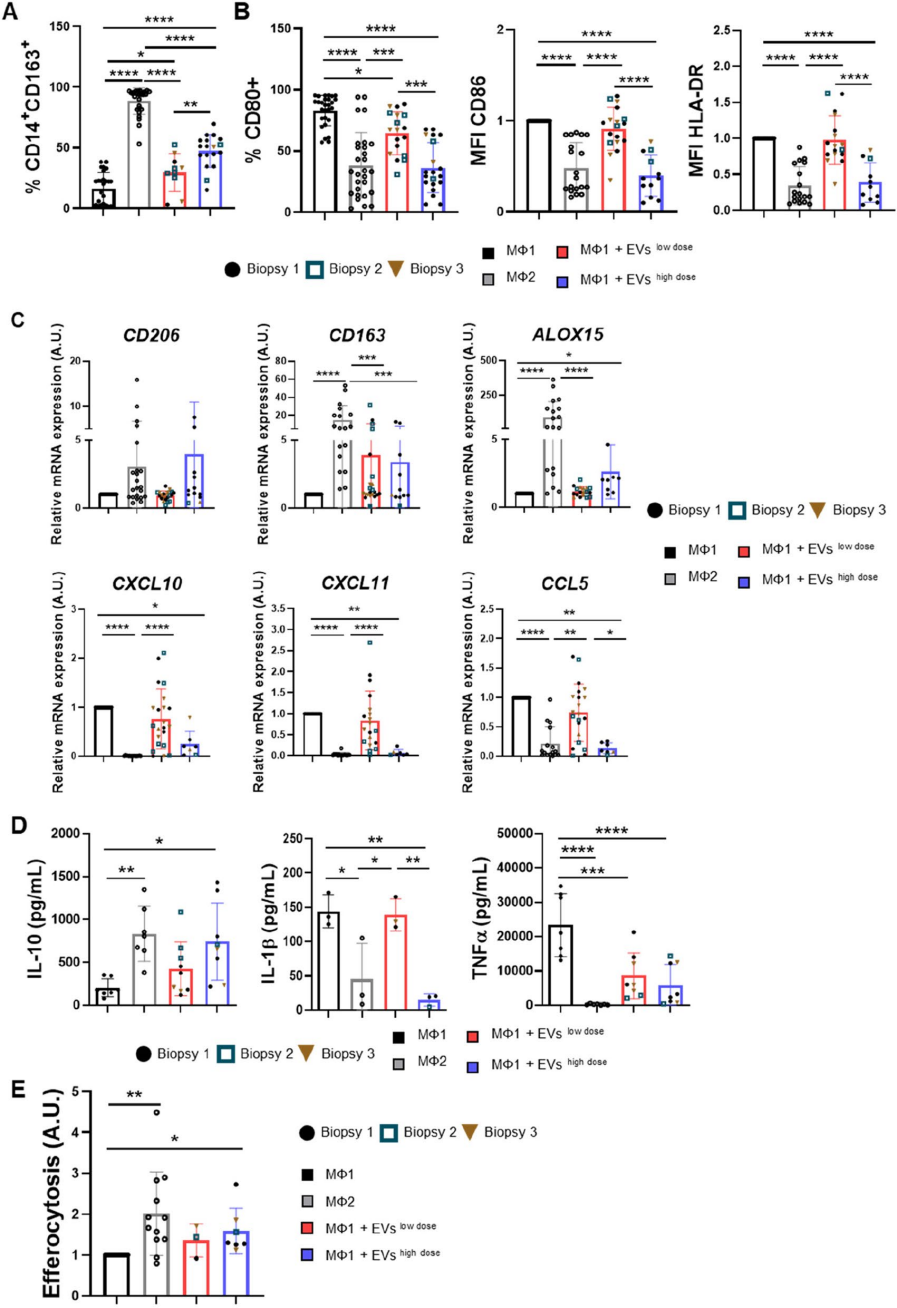
MSC-EVs promote the polarization of M1 macrophages to an M2-like phenotype in a dose-dependent manner. **A** Percentage of CD14^+^CD163^+^ double-positive cells analyzed by flow cytometry at the end of differentiation (*n* ≥ 20). **B** Flow cytometry analysis of CD80, CD86, and HLA-DR markers of M1 macrophages after differentiation. Relative mean fluorescence intensity (MFI) was calculated by dividing all individual data by the mean expression in M1 (*n* ≥ 20). **C** *CD206*, *CD163*, *ALOX15*, *CCL5*, *CXCL10*, and *CXCL11* mRNA expression levels evaluated by RT-qPCR after monocyte differentiation. M1 macrophages were used as the control for normalization (*n* ≥ 7). **D** IL-10 (*n* = 7), IL-1β (*n* = 3), and TNFα (*n* = 7) production was measured by ELISA at the end of differentiation. **E** Quantification of macrophage efferocytic capacity. Apoptotic neutrophils were stained with Texas red dye, and macrophage efferocytic capacity was evaluated by flow cytometry. The graphic shows the MFI of APC-positive macrophages relative to M1 macrophages (*n* ≥ 3). The black bar represents M1 macrophages, the gray bar represents M2 macrophages, and the red and blue bars represent M1 macrophages treated with 2 × 10^9^ particles/ml (low dose, red) and 4 × 10.^9^ particles/mL (high dose, blue) of MSC-EVs, respectively. Graphs represent the mean ± SD of independent experiments. One-way ANOVA with Tukey’s multiple comparisons test was used for statistical analysis of normal data. Kruskal–Wallis with Dunn’s multiple comparisons test was used for statistical analysis of non-normal data (**C** and **D**). Each symbol shape (●, □, ▼) represents a different biopsy of MSC-EVs. *p < 0.05, **p < 0.01, ***p < 0.001, and ****p < 0.0001

Because of their immunomodulatory capacity, MSC-EVs can alter the expression and function of various immune cells [27]. We therefore investigated the immunomodulatory effect of DP-MSC-EVs in an in vitro model of macrophage polarization. Macrophages were differentiated into M1 or M2 phenotypes (see the “Materials and methods” section) and two different doses of MSC-EVs (low dose, 2 × 10^9^; high dose, 4 × 10^9^ particles/mL) were added at the onset of differentiation towards an M1 phenotype. On day 5, M1 and M2 macrophages were activated by the addition of LPS and IFNγ or IL-4 and IL-13, respectively, and the expression of M1 and M2 markers was analyzed by flow cytometry. We assessed the expression of CD163, a classical M2 marker, by determining the percentage of double-positive CD14^+^CD163^+^ cells. We found that the expression of CD163 was significantly higher in EV-treated M1 macrophages than in untreated cells (Fig. 3A). We also evaluated the expression of the M1 markers, CD86, HLA-DR, and CD80 and found significantly lower expression in cells treated with the higher dose of MSC-EVs, reaching levels similar to those of M2 macrophages (Fig. 3B). A comparable effect was found for CD80 expression with the lower dose of MSC-EVs. These results indicate that MSC-EVs can dose-dependently promote the polarization of M1 macrophages towards an M2-like phenotype.

We next examined the gene expression of several cytokines and membrane markers of M1 and M2 macrophages by qPCR (Fig. 3C). Expression of the classical M2 markers *CD206* and *CD163*, increased slightly in M1 macrophages treated with MSC-EVs, although the differences were not significant. We also evaluated the expression of *ALOX15*, a lipoxygenase that catalyzes the incorporation of oxygen into polyunsaturated fatty acids and contributes to the synthesis of specialized pro-resolving mediators (SPMs) [49]. SPMs are chemical mediators that promote the resolution of inflammatory responses. We observed that *ALOX15* expression was higher in M2 macrophages than in M1 macrophages and that treatment of the latter with the higher dose of MSC-EVs resulted in a significant increase in *ALOX15* expression (Fig. 3C). This could indicate a higher capacity for SPM synthesis in MSC-EV-treated macrophages and, therefore, a higher resolution profile than in untreated M1 macrophages. Contrastingly, the expression of the M1 cytokines *CCL5*, *CXCL10*, and *CXCL11* was significantly decreased after treatment with MSC-EVs, reaching expression levels similar to those of M2 macrophages.

We next evaluated IL-10, IL-1β, and TNFα production in EV-treated and untreated macrophages by ELISA. Treatment with MSC-EVs significantly increased the production of the anti-inflammatory cytokine IL-10, similar to the production observed in M2 macrophages. Conversely, the amount of the pro-inflammatory cytokines IL-1β and TNFα was significantly lower in macrophages treated with MSC-EVs than in untreated M1 macrophages (Fig. 3D). Collectively, these results show that MSC-EVs induce a more pro-resolving phenotype, as evidenced by the higher expression of M2 markers and the lower production of pro-inflammatory mediators.

We next sought to test whether these modifications were functional by evaluating the efferocytic capacity of EV-treated and untreated macrophages. Macrophages were co-cultured with dyed apoptotic neutrophils, and we assessed neutrophil capture by flow cytometry. M1 macrophages treated with MSC-EVs resembled the efferocytic capacity of M2 macrophages, which was significantly higher than that of untreated M1 macrophages (Fig. 3E).

Overall, these experiments indicate that macrophages treated with MSC-EVs not only display markers characteristic of M2 macrophages, but also behave functionally similar to them. Therefore, MSC-EVs derived from DP-MSCs could be good therapeutic agents in pathologies involving inflammatory processes, promoting the resolution of inflammation.

### Administration of MSC-EVs in nude rats after acute myocardial infarction reduces infarct size

The ability of MSC-EVs to modulate macrophages towards an M2-like phenotype led us to investigate their effects in a rat model of AMI. Animals were randomized into four groups: two saline groups and two MSC-EV-treated groups. The procedure consisted of permanent ligation of the left anterior descending coronary artery (LAD). A total of 2.5 × 10^9^ MSC-EVs of the three biopsies were administered intramyocardially immediately after the procedure, and their effect was evaluated 7 or 21 days later.

**Fig. 4:**
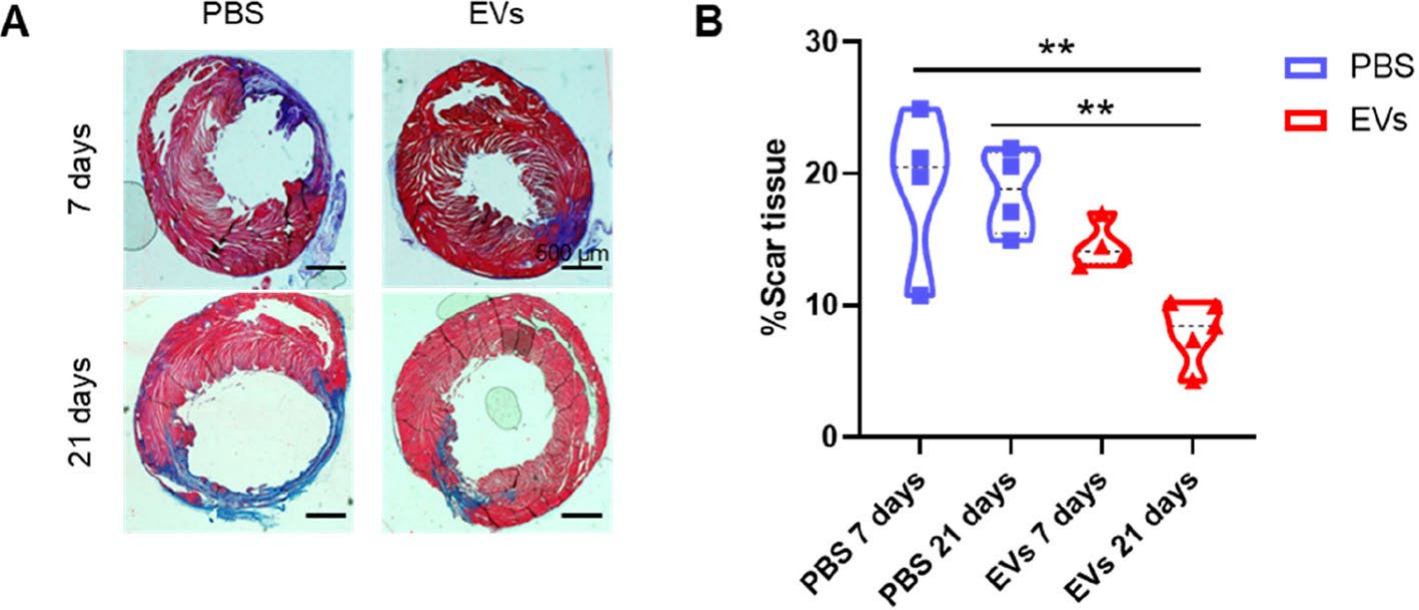
MSC**-**EV administration reduces scar tissue formation in an acute myocardial infarction (AMI) rat model. **A** Representative images of Masson’s trichrome staining were used to quantify the percentage of scar tissue 7 and 21 days after AMI; scale bar = 500 µm. **B** Quantification of the fibrotic area was calculated as the percentage of the blue area divided by the total tissue area (*n* = 4 (PBS) and *n* = 5 (EVs). Graphs represent the mean ± SD of the percentage of scar tissue in the PBS group at 7 and 21 days (blue symbols) and MSC-EV group at 7 and 21 days, respectively (red symbols). One-way ANOVA with Tukey’s multiple comparisons test was used for the statistical analysis of panel **B**. **p < 0.01

The extent of scar tissue after AMI is directly related to an increased likelihood of heart failure. With this in mind, we evaluated the effect of MSC-EVs on scar formation by measuring the fibrotic tissue generated 7 and 21 days after the procedure. Results showed that scar formation was significantly lower in MSC-EV-treated mice than in saline-treated mice at 21 days post-AMI (Fig. 4).

### Genetic and protein profiles of hearts after *AMI* are modified by treatment with MSC-EVs

**Fig. 5:**
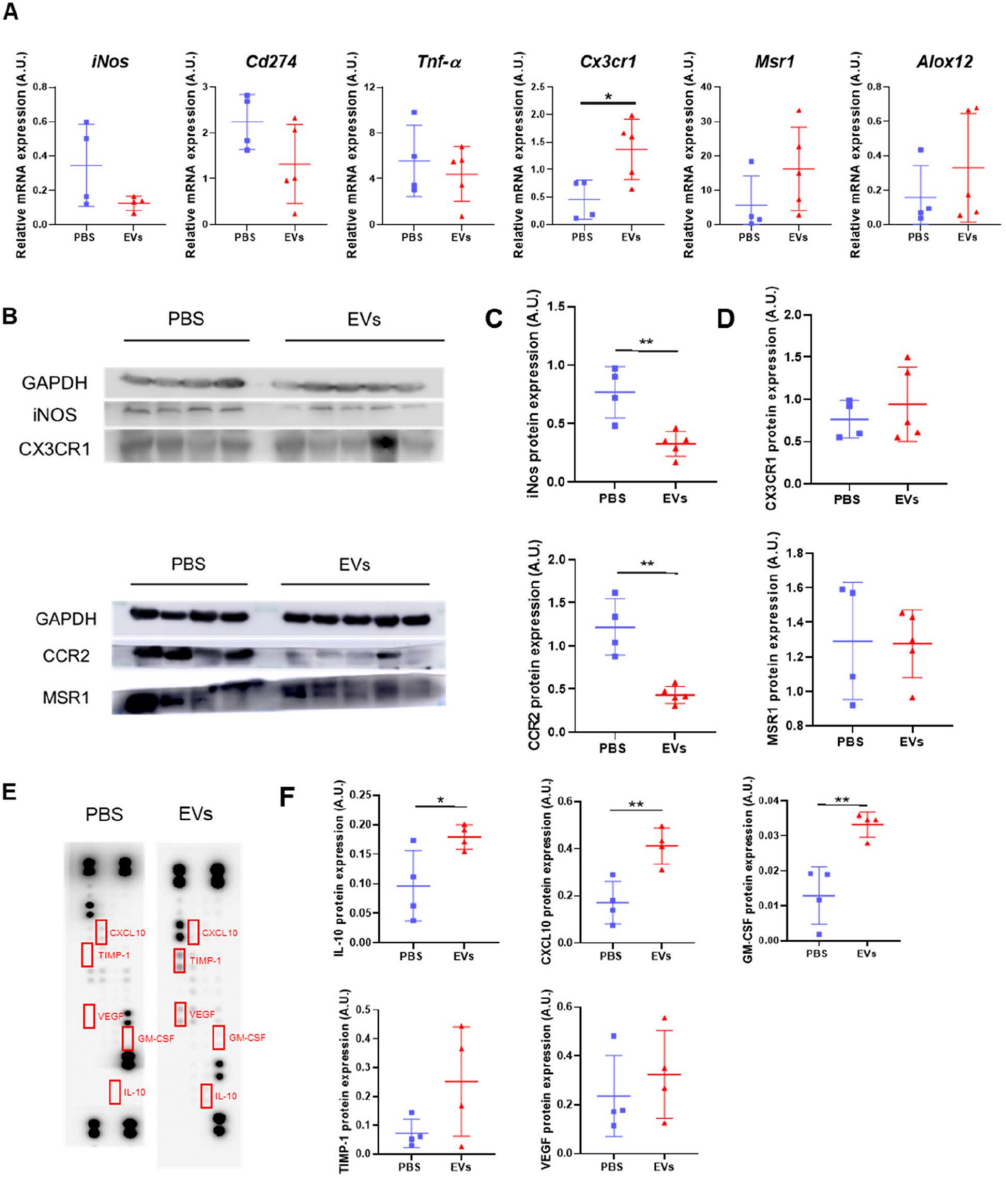
MSC-EV treatment decreases the gene and protein expression of M1 markers in the infarct area. **A** mRNA expression levels of *iNos*, *Cd274*, *Tnf-α*, *Msr1*, *Alox12*, and *Cx3cr1* quantified by RT-qPCR in the infarct area of rat hearts treated with PBS (control, *n* = 4) or MSC-EVs (*n* = 5). **B** Representative western blots of M1 (iNOS and CCR2) and M2 (CX3CR1 and MSR1) markers in infarct heart sections of rats treated with PBS or MSC-EVs. Expression levels were quantified by densitometry, and GAPDH was used as a loading control. **C** Quantification of expression levels of M1 markers iNOS and CCR2. **D** Quantification of expression levels of M2 markers CX3CR1 and MSR1. **E** Representative images of the cytokine array performed in the PBS- and MSC-EV-treated groups. Red boxes represent the cytokines quantified. Cytokine expression levels were quantified by densitometry. **F** IL-10, CXCL10, GM-CSF, TIMP-1, and VEGF quantification (*n* = 4). Graphs represent the mean ± SD. Unpaired *t* tests were used for the statistical analysis. *p < 0.05 and **p < 0.01

Given the evident in vitro effects of MSC-EVs on macrophages, we sought to determine whether in vivo treatment with MSC-EVs promotes faster resolution of inflammation. We, therefore, studied the groups of rats that were euthanized at 7 days post-infarction. We chose this time point because, although the inflammatory peak occurs somewhat earlier, this is the time when the resolution of cardiac inflammation develops and the number of pro-inflammatory cells such as macrophages and neutrophils decreases [50]. We first evaluated the macrophage phenotypes in the infarcted area of control and MSC-EV-treated rats in terms of gene expression. The M1 markers *iNos* and *Cd274* showed a trend for lower expression in EV-treated rats than in saline-treated rats. We also observed that the expression of *Tnfα*, a pro-inflammatory cytokine, was lower in the EV-treated group. Contrastingly, the M2 markers *Cx3cr1* and *Msr1* showed higher expression in the EV-treated group than in the saline-treated group, which was significant for *Cx3cr1* (Fig. 5A). *Alox12* expression was also higher in the rats treated with MSC-EVs (Fig. 5A).

We also evaluated the protein expression of pro-inflammatory and pro-resolving molecules (Fig. 5B). As shown in Fig. 5C, the expression of the M1-type markers iNOS and CCR2 in the infarcted area was significantly lower in EV-treated rats than in saline-treated rats, whereas the protein expression of the M2 markers CX3CR1 and MSR1 was not different between both groups (Fig. 5D), in contrast to the gene expression.

Finally, we used a cytokine array to simultaneously determine the protein expression of multiple cytokines in the infarct area (Fig. 5E). We used samples from four rats of each group (saline- and EV-treated). The only cytokines that showed significant differences were IL-10, CXCL10, and GM-CSF (Fig. 5F), all of which were higher in the rats treated with MSC-EVs. Other differences in cytokine levels were noted between both the groups, such as TIMP1 and VEGF, which showed a trend for increased expression in the group of rats treated with MSC-EVs, although the increase was not significant.

## MSC-EV treatment reduces the abundance of M1 macrophages in the infarcted area

**Fig. 6:**
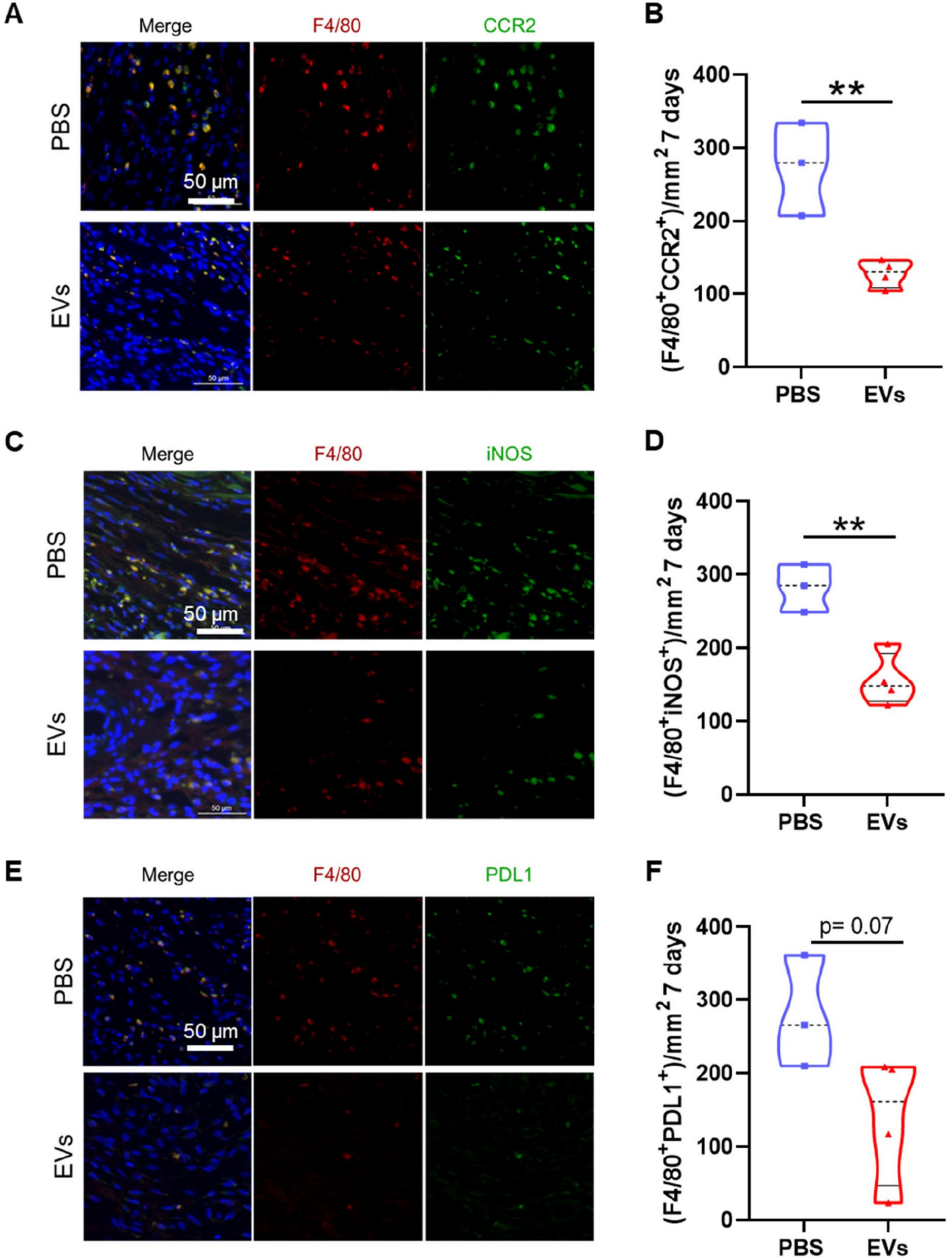
MSC-EVs reduce the amount of infiltrating M1 macrophages in the infarcted area of animals sacrificed at 7 days. **A** Representative images of immunodetection of F4/80^+^ (general macrophage marker, red) and CCR2^+^ (M1 marker, green) in the infarcted area of heart Sects. 7 days after AMI. Nuclei were stained with DAPI (blue). Scale bar = 50 µm. **B** Quantification of F4/80^+^CCR2^+^ double-positive cells per mm^2^ in the infarcted area of rats treated with PBS or MSC-EVs 7 days after AMI. **C** Representative images of immunodetection of F4/80^+^ (red) and iNOS (M1 marker, green) in the infarcted area of heart Sects. 7 days after AMI. Nuclei were stained with DAPI (blue). Scale bar = 50 µm. **D** Quantification of F4/80^+^iNOS^+^ double-positive cells per mm^2^ in the infarcted area of rats treated with PBS or MSC-EVs 7 days after AMI. **E** Representative images of immunodetection of F4/80^+^ (red) and PDL1 (M1 marker, green) in the infarcted area of heart Sects. 7 days after AMI. Nuclei were stained with DAPI (blue). Scale bar = 50 µm. **F** Quantification of F4/80^+^PDL1^+^ double-positive cells per mm.^2^ in the infarcted area of rats treated with PBS or MSC-EVs 7 days after AMI. PBS group, *n* = 3; MSC-EV group, *n* = 4. Graphs represent the mean ± SD. Unpaired *t* tests were used for the statistical analysis. *p < 0.05 and **p < 0.01

We next evaluated the populations of macrophages in the infarcted rat hearts at the histological level to determine the extent of macrophage modulation after treatment with MSC-EVs. We performed immunofluorescence analysis of different M1 and M2 markers in combination with the general macrophage marker F4/80. At 7 days post-AMI, the number of M1 macrophages infiltrated in the infarct area, analyzed as F4/80^+^CCR2^+^ (Fig. 6A, B), F4/80^+^iNOS^+^ (Fig. 6C, D), and F4/80^+^PDL1^+^ (Fig. 6E, F), was lower in EV-treated rats than in saline-treated rats, which was significant for CCR2 and iNOS. The M2 macrophage population was analyzed by studying double-positive cells for F4/80^+^CX3XR1^+^, F4/80^+^MSR1^+^, F4/80^+^ARG1^+^, and F4/80^+^CD206^+^ (Supplementary Fig. 1). Unlike M1 macrophages, no differences were observed for M2 macrophages between the groups. Some M2 markers such as CX3CR1 and MSR1 showed similar levels of expression between groups, whereas ARG1 and CD206 were expressed to a lower extent in the MSC-EV-treated group.

We repeated this analysis 21 days post-AMI (Supplementary Fig. 2). F4/80^+^PDL1^+^ and F4/80^+^CCR2^+^ macrophages (M1) were slightly decreased in the infarct area of rats treated with MSC-EVs, whereas the amount of F4/80^+^CD206^+^ and F4/80^+^CX3CR1^+^ macrophages (M2) was similar in both groups.

Taken together, our results show that treating AMI with MSC-EVs promotes the reduction in the number of M1 macrophages, likely creating a less inflammatory and more pro-resolving environment. The faster resolution of inflammation could favor recovery after the AMI.

## Discussion

 Cell therapy is an emerging strategy for AMI and has already shown beneficial effects on several pathologies. Because an inflammatory response is a characteristic pathological feature of AMI [51], MSCs have been considered good cell therapy candidates on the basis of their immunomodulatory capacity [52]. Although the treatment of inflammation has received little attention in the context of AMI, recent studies have shown that targeting different components of the inflammatory process can be an effective therapeutic approach to reduce the long-term negative consequences of AMI.

The beneficial effects of MSCs are mainly attributed to their paracrine actions, which are primarily mediated by secreted EVs. Although EVs derived from different tissue sources have distinct properties, it has been demonstrated that there is also variability in the EVs obtained from the same tissue source from different individuals. For example, Baglio et al. showed that the non-coding RNA content of MSC-EVs was tissue-dependent and influenced their therapeutic properties [53]. Additionally, the miRNA content of EVs is influenced by the age of the donor [35]. As the number of EVs needed for treatment is very large, obtaining a sufficient number from a single biopsy can be challenging. Accordingly, one of the objectives of the present study was to compare EVs obtained from three different biopsies of DP-MSCs from young healthy donors in terms of their physicochemical characteristics, protein, lipid, and miRNA content and functionality. Characterization of MSC-EVs by NTA, DLS, and electron microscopy revealed a size distribution of 50–200 nm in all MSC biopsies, which is consistent with the standard size for EVs according to ISEV guidelines [54]. These techniques also confirmed that the different sourced MSC-EVs have the same morphology and physicochemical composition as indicated by typical markers, regardless of the biopsy. The proteins present in all MSC-EV samples were involved in biological processes related to extracellular matrix organization, transport, adhesion, and immune response. These findings align with proteomic studies of MSC-EVs from different tissue sources [55, 56]. Likewise, the lipidomic analysis revealed that the lipid composition of MSC-EVs obtained from the different biopsies was consistent, suggesting that the lipid content remains stable when the cell source has the same tissue origin. DP-MSC-EVs contained high levels of phosphatidylcholine and sphingomyelin, as previously described by Lai et al. in their study on exosomes from the conditioned medium of human embryonic stem cell-derived MSCs [57].

Our miRNAseq studies detected 99 miRNAs shared between all three biopsies, regardless of their abundance. Notably, approximately half of these miRNAs have previously been identified in EVs from bone marrow, adipose tissue, or umbilical cord MSCs, suggesting a common miRNA cargo in EVs from MSCs [53, 58, 59]. Among these miRNAs, we observed the presence of miR-210, miR-223-3p, and let-7c-5p, which are noted for their ability to induce polarization towards an M2 phenotype in macrophages [60, 61]. Also of interest was the presence of miR-451a, which showed the highest expression. This miRNA has been related to antioxidant activities and cardio-protection [62, 63].

We have previously described the presence of miR-4732-3p in DP-MSC-EVs with cardioprotective functions [25]. The isolation method used in the present study (TFF-SEC) did not allow for the identification of this miRNA; however, it is important to highlight that miR-451 shares a polycistronic promoter with miR-4732-3p and miR-144 [64]. Further studies will be necessary to elucidate the relationship between these two miRNAs in terms of their presence in DP-MSC-EVs and their therapeutic potential.

Another main objective of this study was to determine the effect of MSC-EVs on the immune processes that occur during AMI. Several studies have shown that MSC-EVs can polarize macrophages from a pro-inflammatory or M1 phenotype to a pro-resolving or M2 phenotype [28, 65]. In all these cases, EVs were obtained from bone marrow or adipose tissue. In the present study, we observed that EVs derived from the three DP-MSC biopsies could repolarize pro-inflammatory macrophages towards an M2-like phenotype, which was characterized by an increase in CD163 expression and a decrease in CD80, CD86, and HLA-DR expression. Molecular analysis also demonstrated that the treatment of macrophages with MSC-EVs induced an M2-like phenotype. The functionality of the M2-like macrophages was confirmed with an efferocytosis assay. Efferocytosis is crucial in the initial stages following AMI to induce the resolution of inflammation [66].

The inflammatory response following AMI plays a role in eliminating damaged cells and extracellular matrix from the ischemic area, as well as in the formation of a new extracellular matrix to create the infarct scar [3, 67]. It has been reported that the size of the infarct and the quality of the scar are directly related to the inflammatory process [3]. An exaggerated inflammatory response can result in a greater extension of the infarcted area and adverse cardiac remodeling, which increases the likelihood of heart failure. Our *in vivo* studies showed that DP-MSC-EVs promote smaller infarct sizes than the saline-treated control group at both 7 and 21 days, with a significant reduction at 21 days. This is consistent with the study by Zhao et al., who also reported the ability of umbilical cord MSC-EVs to reduce infarct size [68].

We analyzed macrophage infiltration in the infarct zone at 7 and 21 days after AMI. Treatment with MSC-EVs resulted in a decrease in the number of M1 macrophages at 7 days after AMI as compared with the control group. This finding is consistent with a study by Sun et al., who used EVs derived from umbilical cord MSCs [65], although their analysis was performed 3 days after AMI. These authors also noted an increase in the number of infiltrating M2 macrophages, but only EVs from 3D MSC cultures were used [69]. We also found that MSC-EV treatment modified the gene expression of pro- and anti-inflammatory markers in the infarct zone. Specifically, there was a decrease in the expression of *inos* and *cd274*, two markers of M1 macrophages, while the gene expression of M2-like macrophage markers such as *cx3cr1* increased. We also found higher gene expression of *alox12*, which is responsible for synthesizing pro-resolving lipid mediators (SPMs). In macrophages, ALOX12 participates in the synthesis of maresins, a family of SPMs that has been associated with the polarization of macrophages towards a pro-resolving phenotype [70] and that, in general, contributes to the resolution of inflammation by acting on different cell types.

We surveyed the inflammatory environment of the infarcted heart by analyzing the protein expression of various cytokines. The experiment yielded unexpected results. Treatment with MSC-EVs led to an increase in IL-10, an anti-inflammatory cytokine secreted by M2 macrophages that favors the resolution of inflammation and repair of the infarcted area. However, there was also an increase in the expression of the cytokines GM-CSF and CXCL10, both historically considered proinflammatory cytokines. GM-CSF is secreted by various cell types, primarily by cardiac fibroblasts, but the role of this cytokine in AMI is complex. GM-CSF has been observed to interact directly with endothelial cells, promoting angiogenesis, likely by stimulating the production of angiogenic compounds such as VEGF [71]. Additionally, GM-CSF has been shown to promote monocyte and neutrophil infiltration in the ischemic zone [72] and can improve collateral flow in patients with coronary artery disease [73]. A study in patients with AMI demonstrated that GM-CSF administration led to improvements in ventricular function after 12 months [74]. Therefore, increased GM-CSF levels may have both beneficial and detrimental effects, depending on the extent to which it exacerbates the inflammatory response. CXCL10 was moderately increased in the group of animals treated with MSC-EVs. The role of CXCL10 in AMI is also complex. While it has been linked to increased recruitment of immune cells during the initial days following infarction, it has also been observed to have angiostatic and antifibrotic effects on endothelial cells and fibroblasts, respectively [75, 76]. This could potentially impede neovascularization and premature fibrosis before the removal of all apoptotic cellular debris from the damaged area. The mechanisms underlying the actions of these cytokines remain unclear and require further elucidation.

Our study has several limitations that should be considered. First, we used the M1/M2 macrophage classification to assess the immunological impact of MSC-EVs on infarcted rat hearts. Although this paradigm provides a useful framework for understanding macrophage polarization in response to cardiac injury, recent research by Walter et al*. *[14] and Nahrendorf et al. [77] suggest that the actual landscape of macrophage phenotypes in the heart, particularly post-injury, is more complex and does not strictly adhere to this dichotomy. These studies emphasize the presence of macrophages with mixed or transitional activation states, challenging the oversimplification of the M1/M2 classification in capturing the dynamic and nuanced roles of macrophages in myocardial repair and inflammation. Also, Alonso-Herranz et al. [78] have highlighted the complex interplay between macrophages and cardiac repair, emphasizing the need for a refined understanding of macrophage phenotypes beyond the traditional M1/M2 polarization. Second, we utilized a rat model of myocardial infarction, which differs from the more commonly used mouse models in cardiac research. Macrophage populations in rats are not as thoroughly characterized as those in mice, particularly regarding the specific antigens that define M1 and M2 macrophage subsets. Third, our study employed nude rats, which lack T cell-mediated adaptive immune responses, to investigate the therapeutic effect of MSC-EVs. While these animals maintain innate immunity, their lack of T cell responses poses an additional limitation to our study. We anticipate that the macrophage population should remain unaffected by the absence of T cell activity; however, this unique aspect of the immune system in nude rats may influence the overall immune response to myocardial infarction and the interaction with MSC-EVs. These considerations underscore the need for a cautious interpretation of our findings and suggest the need for further studies to understand the mechanisms of action of MSC-EVs during cardioprotective therapies.

## Conclusions

In conclusion, our study highlights the therapeutic potential of DP-MSC-EVs in modulating macrophage activity towards a reparative role in the context of AMI. However, it is important to acknowledge the limitations of our study, particularly the simplistic binary classification of macrophages and the unique immune context of the nude rat model, which lacks T cell responses. The findings indicate that DP-MSC-EVs are potentially a valuable tool for cardiac regeneration; however, further research is needed to fully understand their mechanism of action in diverse immunological settings.

### Supplementary Information

Additional file 1: Table S1. Excel file showing the lipidomic analysis to identify the lipid groups in the three biopsies of MSC-EVs.

Additional file 2: Table S2. Excel file showing the identified proteins from the 3 different MSC-EVs biopsies classified by the unused value.

Additional file 3: Table S3. Excel file representing read counts of 99 common miRNAs in MSC-EVs from the three biopsies.

Additional file 4: Figure S1. Quantification of double-positive F4/80 + CX3CR1 + (A), F4/80 + MSR1 + (B), F4/80 + ARG1 + (C), and F4/80 + CD206 + (D) M2 macrophages per mm^2^ in the infarcted area of rats treated with PBS or MSC-EVs 7 days after AMI.

Additional file 5: Figure S2. Quantification of double-positive F4/80 + PDL1 + , F4/80 + CCR2 + (A) for M1, F4/80 + ARG1 + , and F4/80 + CD206 + (B) for M2 macrophages per mm^2^ in the infarcted area of rats treated with PBS or MSC-EVs 21 days after AMI.

Additional file 6: Table S4. Excel file showing miRNAs and proteins related to each of the biological processes set out in Table 1 and Fig. 2B.

## Data Availability

All data generated or analyzed during this study are included in this published article and its supplementary information files. Raw lipidomic data is available upon request to the corresponding author. The miRNAseq data is available in the GEO database (http://www.ncbi.nlm.nih.gov/geo/) and the accession number GEO: GSE260506.

## Abbreviation

miRNA: Microribonucleic acid
MSC: Mesenchymal stromal cell
PFA: Paraformaldehyde
HBSS: Hank’s Balanced Salt Solution
GO: Gene ontology
EVs: Extracellular vesicles
AMI: Acute myocardial infarction
SPMs: Specialized pro-resolving lipid mediators
TFF: Tangential flow filtration
SEC: Size exclusion chromatography
UC: Ultracentrifugation
