# Supplementary figures and images for: Extracellular vesicles from dental pulp mesenchymal stem cells modulate macrophage phenotype during acute and chronic cardiac inflammation in athymic nude rats with myocardial infarction
Source: Inflamm Regen. 2024 May 28;44:25. doi: 10.1186/s41232-024-00340-7 (PMC11134765; doi:10.1186/s41232-024-00340-7)

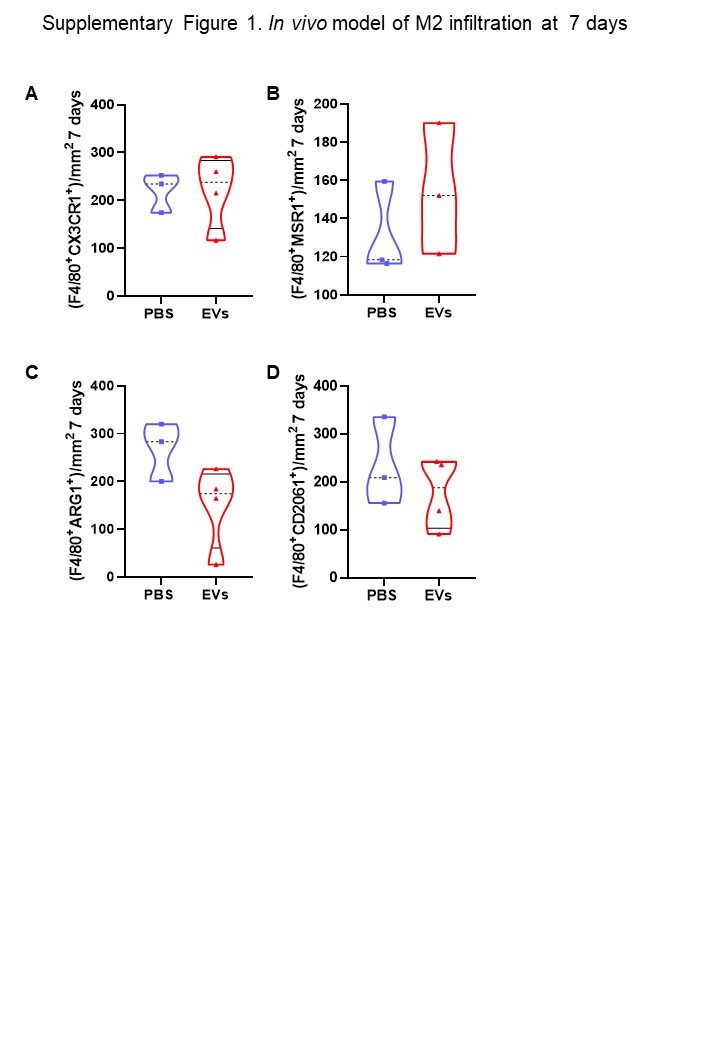

Supplement: Supplementary file 4 — Additional file 4: Figure S1. Quantification of double-positive F4/80 + CX3CR1 + (A), F4/80 + MSR1 + (B), F4/80 + ARG1 + (C), and F4/80 + CD206 + (D) M2 macrophages per mm2 in the infarcted area of rats treated with PBS or MSC-EVs 7 days after AMI. [file 41232_2024_340_MOESM4_ESM.jpg]

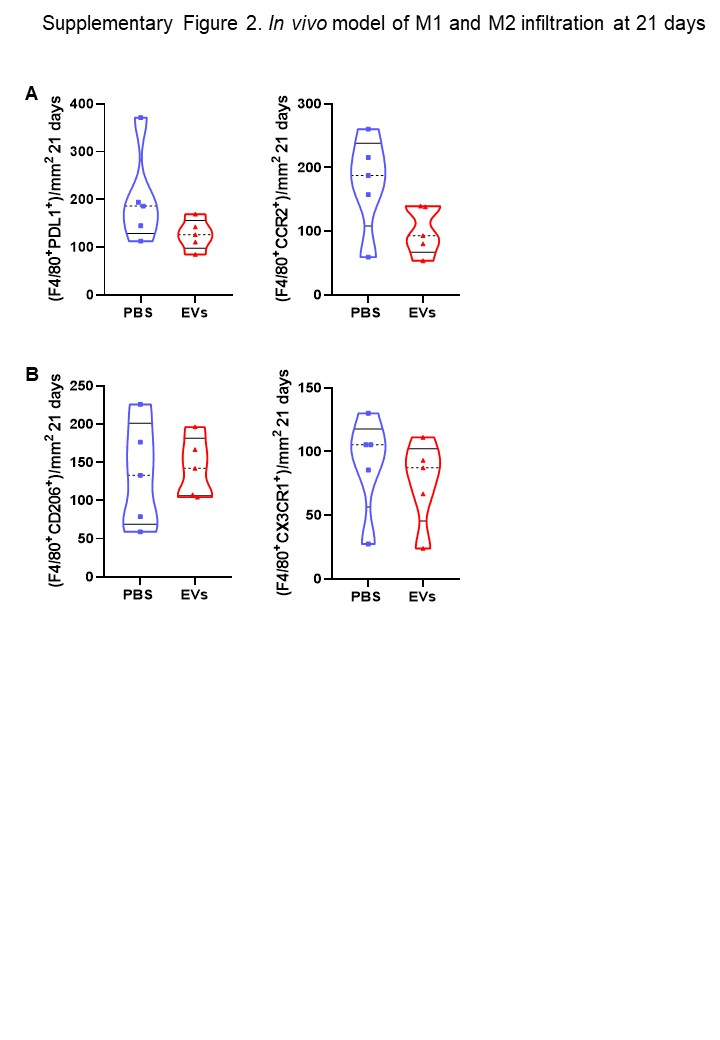

Supplement: Supplementary file 5 — Additional file 5: Figure S2. Quantification of double-positive F4/80 + PDL1 + , F4/80 + CCR2 + (A) for M1, F4/80 + ARG1 + , and F4/80 + CD206 + (B) for M2 macrophages per mm2 in the infarcted area of rats treated with PBS or MSC-EVs 21 days after AMI. [file 41232_2024_340_MOESM5_ESM.jpg]
